# Seasonal and Spatial Variations of Indoor Pollen in a Hospital

**DOI:** 10.3390/ijerph6123169

**Published:** 2009-12-10

**Authors:** Rafael Tormo-Molina, Ángela Gonzalo-Garijo, Inmaculada Silva-Palacios, Santiago Fernández-Rodríguez

**Affiliations:** 1 Botany Area, Science Faculty, University of Extremadura, Badajoz, Spain; E-Mail: santiferro@unex.es; 2 Department of Allergology, Infanta Cristina University Hospital, Badajoz, Spain; E-Mail: magonzalog@telefonica.net; 3 School of Agrarian Engineering, University of Extremadura, Badajoz, Spain; E-Mail: insilva@unex.es

**Keywords:** bioaerosols, airborne pollen, allergy, hospital, aerobiology

## Abstract

The airborne indoor pollen in a hospital of Badajoz (Spain) was monitored over two years using a personal Burkard sampler. The air was sampled in four places indoors—one closed room and one open ward on each of the ground and the third floors—and one place outdoors at the entrance to the hospital. The results were compared with data from a continuous volumetric sampler. While 32 pollen types were identified, nearly 75% of the total counts were represented by just five of them. These were: *Quercus*, Cupressaceae, Poaceae, *Olea*, and *Plantago*. The average indoor concentration was 25.2 grains/m^3^, and the average indoor/outdoor ratio was 0.27. A strong seasonal pattern was found, with the highest levels in spring and winter, and the indoor concentrations were correlated with the outdoor one. Indoor air movement led to great homogeneity in the airborne pollen presence: the indoor results were not influenced by whether or not the room was isolated, the floor level, or the number of people in or transiting the site during sampling. The presence of ornamental vegetation in the area surrounding the building affected the indoor counts directly as sources of the pollen.

## 1. Introduction

Indoor bioaerosols have been investigated worldwide, mainly because we humans now spend most of our time indoors, the fraction for industrialized countries ranging from 80% [1,2] to 95% [3]. These studies have mainly been concerned with airborne fungi, while the indoor airborne presence of pollen grains has received less attention. Furthermore, pollen allergens in indoor air could occur predominantly as particles of smaller diameter than pollen grains [4].

Although the term “sick building syndrome” has been used to denote the origin of indoor allergic and environmental illnesses, the main biological causes of building-related disorders are fungi, bacteria, viruses, protozoa, pollen, house-dust mites, insect pests, algae, pigeons, and rodents [5,6]. It is recognized that airborne allergies cause more problems worldwide than all other allergies combined. Furthermore, exposure to substances found in the indoor environment is known to trigger such respiratory illnesses as asthma. Studies of the association between human illness and environmental exposure focused initially on the outdoor environment in the form of pollen and mould counts. The emphasis has now shifted, however, to exposure in indoor environments [7,8], and although pollens as bioaerosols are often not considered in air pollution studies their concentration may measured in air quality standards [9].

Different proposals have been made to reduce or eliminate indoor bioaerosols, with efficiencies close to 100%, basically by filtering the air with electrostatically charged activated carbon [4,10] or using HEPA (high efficiency particulate air) filters [11,12]. Nevertheless, it has also been argued that the simple use of air-conditioning can reduce indoor bioaerosols [13].

Most indoor airborne pollen research has been on homes [13–19], including mobile homes [3]. Other contexts that have been investigated are workplaces [4,20] and schools [4]. Work on airborne pollen in hospital environments has been less frequent [21].

Most researchers agree that there is a close relationship between outdoor and indoor pollen, although there are some discrepancies [14,20]. There are various possible routes by which pollen may enter the indoor airborne environment, such as through the ventilation system [22], but there is a trend of thought that humans themselves are the vector, either transporting these bioaerosols [23] adhered to their clothing [16] or stirring them up from the floor where they are abundant, as people walk about inside a building [19,24–26].

Although there are techniques than permit sampling allergens in the air [27] quantification of indoor airborne pollen continues as accurate method of study. Various samplers have been used to detect indoor airborne pollen. Gravimetric samplers were commonly used initially, but have progressively declined in popularity [28–31]. Personal volumetric samplers are extensively used, examples being the Rotorod [3,15,19,32], the button inhalable aerosol sampler [33], Lanzoni [20,21], and the Burkard personal sampler [17,22]. In some cases, a fixed or continuous sampler has been used to compare indoor and outdoor sampling data, examples being the Burkard seven-day [18] and the Lanzoni [20] samplers.

The aim of the present work was to sample the indoor atmosphere of a hospital to determine the temporal and spatial variation in pollen concentrations, to compare these concentrations with outdoor measurements, and then to assess the possible factors that might influence the presence of pollen, such as the level of isolation of the room, the number of people transiting the room, the weather, and seasonality.

## 2. Material and Methods

### 2.1. Study Area and Pollen Sampling Methods

Sampling was carried out in the Hospital Infanta Cristina in Badajoz (Spain). This is an eight-storey building on the outskirts of the city. The sampling period began in April 2007 and ended in March 2009. A total of 60 samples were taken, weekly in three months (April to June, 24 samples) and fortnightly during the rest (July to March, 36 samples), more intensively in spring because is the season when airborne pollens reach the highest concentrations in Mediterranean countries. A personal Burkard sampler was used, with slides with white petrolatum as adhesive. The sampler was placed directly on the floor (a previous study had shown no statistically significant differences between results at floor level and those at a height of one metre [34]). Data from a continuous seven-day Burkard sporetrap [35] were used to make comparisons. This sampler was located in the Agrarian Engineering School of the University of Extremadura in Badajoz (SW Spain, Lat 38.89° Long −6.97°), on a roof terrace at 6 m above ground level at 2.9 km from the hospital.

One outdoor and four indoor sites were selected. The outdoor site was near the main patients’ entrance to the hospital. The indoor sites were located on the ground and the third floors of the building. In both cases, an open ward and a closed room were selected. On the ground floor, the area of the open ward was 148 m^2^, and that of the closed room 17 m^2^ (without windows). On the third floor, the open ward was 49 m^2^, and the closed room 9 m^2^ (with a window closed most of the time). For each open ward and the outdoor entrance, a count was made of the number of people seated or in transit during the sampling period. During the outdoor sampling, the number of vehicles at the entrance was also counted. Sampling was performed in the morning between 10:00 h and 12:00 h for ten minutes with Burkard samplers. The sporetraps have an intake air flux of 10 litres/minute, so that a total of 0.1 m^3^ of air was sampled. The slides were covered with glycerogelatin, and all the pollen grains in the 14 mm long deposition line were counted under 400× magnification light microscopy. The Regional Meteorological Centre provided weather parameters; the meteorological station was located 0.7 km from the hospital studied and 3.2 km from the continuous spore trap station, daily data when sampling in the hospital were done were used in the comparisons.

### 2.2. Statistical Analysis

The Shapiro-Wilk test was used to evaluate the normality of the data. An ANOVA was performed to test for differences by season, year, or site. The Pearson correlation coefficients (r) of the monthly pollen data with the meteorological data (mean temperature, rainfall, wind speed, and relative humidity) were calculated, and with the number of people present in the wards during the sampling period and the number of vehicles at the outdoor entrance.

## 3. Results

### 3.1. General Values of Pollen Concentrations

**Figure 1.**
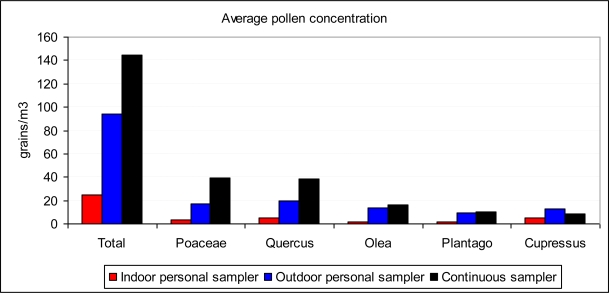
Average pollen concentration, total and for the main five pollen types, using indoor and outdoor personal samplers or continuous sampler.

The number of pollen types identified was 32. The most frequent were, in decreasing order: Poaceae, *Quercus*, *Olea*, *Plantago*, and Cupressaceae, representing 74.4% of the total pollen count (Figure 1). Total pollen concentrations varied over the course of the year, with maxima in spring. The outdoor maximum concentration was 730 grains/m^3^ (24 April 2007) using the personal sampler. For indoor sampling, the maximum was 470 grains/m^3^ in the third-floor closed room (6 February 2008). The average concentration using the personal sampler was 94.5 grains/m^3^ outdoors and 25.1 grains/m^3^ indoors for all the samples, and 144.4 grains/m^3^ for the continuous sampler. With these figures, the average indoor/outdoor ratio (I/O) was 0.27 using data from the same sampler. This value was different for each pollen type: *Quercus* (0.28), Cupressaceae (0.41), Poaceae (0.22), *Plantago* (0.19), and *Olea* (0.14). Despite the average outdoor concentration measured with the continuous sampler being higher than with the personal sampler (53% higher with the respective daily values given above as measured for the same days), the contrary was the case for the outdoor Cupressaceae concentrations, with the personal sampler giving higher values than the fixed sampler.

### 3.2. Temporal Variations in Pollen Concentration

**Figure 2.**
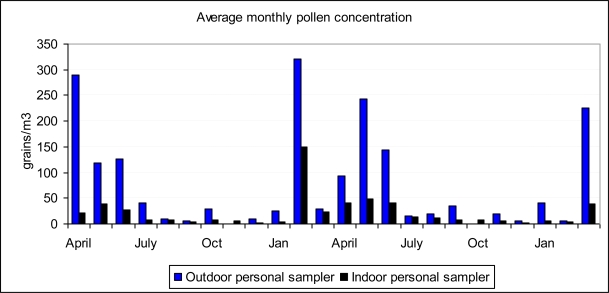
Average monthly indoor and outdoor pollen concentrations from April 2007 to March 2009.

The monthly variation of the indoor and outdoor concentrations is shown in Figure 2. In this case, the I/O rate varied between 0.075 and 0.917, although on two occasions the measured outdoor pollen monthly concentration was zero. The inclusion of correspondingly very low values would have made the summer ratio somewhat greater. The months February to June are those with the highest concentrations, depending on the year.

**Figure 3.**
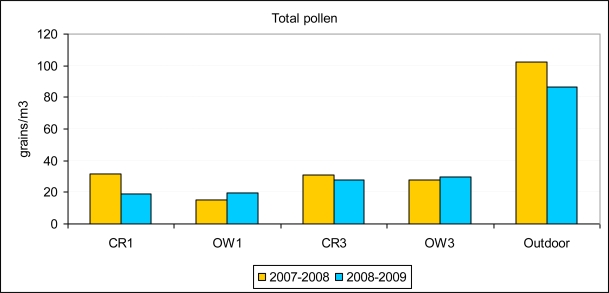
Average annual pollen concentrations outdoors and in the four indoor sites sampled (CR1 closed room ground floor, OW open ward ground floor, CR3 closed room third floor, OW3 open ward third floor).

The data were not normally distributed (Shapiro-Wilk 0.705, p-value < 0.000), but were so after applying a logarithmic transformation (log-normal, Shapiro-Wilk 0.975, p-value 0.518). The ANOVA comparing total pollen data between the two years showed them to be statistically indistinguishable (F 0.136, p-value 0.714). Figure 3 shows this in relation to the five sites sampled. Neither did the same analysis for the five main pollen types separately show any difference between years.

**Figure 4.**
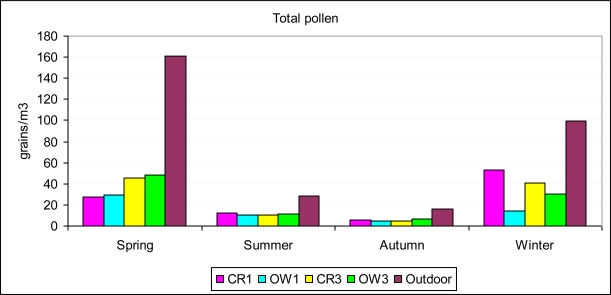
Average seasonal pollen concentration in the five places sampled (see legend to Figure 3).

There were major differences between seasons, with the highest values in spring and winter (F 17.869, p-value < 0.000). Figure 4 shows these differences for each sampling site. For *Plantago* and *Olea* the values were nearly zero in summer, autumn, and winter, and for the rest they were very low, except for Cupressaceae in winter.

### 3.3. Comparisons between Sampling Sites

The ANOVA comparing the five sites showed no differences (F 1.904, p-value 0.132). In view of the strong seasonality, with many zero values, the analysis was repeated with data from only five months (February to June). In this case, there appeared a statistically significant difference (F 5.663, p-value < 0.000), and post-hoc HDS Tukey comparisons showed that the outdoor data were indeed different from the indoor ground floor data.

There was a strong correlation between the indoor and the outdoor data (p-value 0.000 in all cases). This correlation was higher using the data from the two open wards (r 0.529 and r 0.621, ground and third floors, respectively). There was a significant correlation between the data from the two outdoor samplers, continuous and personal (r 0.566, p-value < 0.000). The ANOVA comparing those data showed no statistically significant differences (F 0.426, p-value 0.517), even for the five main pollen types. Nevertheless the concentrations measured with the continuous sampler were higher than with the personal sampler.

### 3.4. Correlation with Weather Parameters, People and Vehicles

The correlation analysis with the weather parameters showed no significant relationships. The correlation analysis for the two open wards and outdoors with the number of people present during sampling only gave a statistically significant correlation for the case of the outdoor data (r 0.353, p-value 0.030). No correlation was found of the outdoor data with the number of vehicles present.

## 4. Discussion

The average I/O ratio found in the present work (0.27) is higher than most values reported in the literature: 0.02 [19,25], 0.025 [33], 0.041 [24], 0.05 [15,20], 0.083 [29], and 0.24 [3]. But it is lower than one case: 0.33 [14]. These differences could be because the present study corresponded to a place in which there is a great movement of people, and the entrance doors are open much of the time. The summer I/O ratio was the highest (0.39), and the spring ratio the lowest (0.23). This is coherent with the findings of Sterling and Lewis [3], who suggested that an explanation for their results could be that air-conditioning, which is most often used in summer, is an entrance route for pollen, or that windows and doors were more likely to be open, thus permitting the entrance of pollen.

The indoor pollen concentration found (25.1 grains/m^3^) is also high compared to most literature values: 1.2 grains/m^3^ [15,19], 10 grains/m^3^ [32], 16 grains/m^3^ [18]. But it is lower than one case: 71 grains/m^3^ [33]. The vegetation surrounding the places sampled in those papers is not reported, but in our case there were abundant ornamental trees and shrubs, mainly Cupressaceae (*Platycladus orientalis, Cupressus arizonica, C. sempervirens*), which would be the origin of the main pollen types found. One must therefore accept that major mixing of indoor and outdoor air is occurring, which is one of the most serious problems of indoor air quality [36].

Like other workers [18,20], we found that the concentrations measured with the continuous sampler were higher than with the personal sampler. This could be because the outdoor site selected for sampling with the personal sampler was at the entrance to the hospital, and protected from the wind. The resulting relative lack of air circulation could therefore have reduced the presence of pollen. As was mentioned above, the exception was the ornamental Cupressaceae, for which the outdoor concentration measured with the personal sampler was higher than with the fixed sampler. A phenological study was conducted for this pollen type in the period 2008–2009 (unpublished data), finding that the highest indoor concentration found in the hospital coincided with the maximum peak for *Platycladus orientalis*, the most abundant ornamental tree around the hospital, in February 2008.

The correlation found between the number of people moving through the entrance and the outdoor pollen counts would seem to support the idea that the pollen is transported by people walking [19,24,25,26] or by a re-suspension process that depends directly on particle size [37]. However, the outdoor sampling was made about ten metres away from the entrance, so that only simultaneous sampling immediately outside the entrance could support or refute this idea. Furthermore, no correlation was found between the number of people present in the wards and the pollen count.

## 5. Conclusions

Although people could be the vectors responsible for a certain amount of indoor pollen carried from outdoors to indoors, we consider mainly the doors and windows, and then the air-conditioning system, to have been the principal routes of the entrance of pollen. The indoor pollen concentrations reached somewhat more than a quarter of the outdoor concentration, with peaks as high as some hundreds of pollen grains per cubic metre on some occasions. This pattern depended on the vegetation surrounding the building—mainly ornamental trees and shrubs. Indoor air circulation could have been the reason for there being no differences between indoor open wards and isolated rooms, and even between independent floors. The outdoor pollen seasonality was also observed indoors, as there was a close correlation in the presence of airborne pollen between the two sets of places. Air-conditioning can alter the I/O ratio either directly as a route for the entry of pollen, or because its use affects the likelihood of doors and windows being closed or open.
